# Gender dimorphism and age of onset in malignant peripheral nerve sheath tumor preclinical models and human patients

**DOI:** 10.1186/1471-2407-14-827

**Published:** 2014-11-15

**Authors:** Elizabeth Shurell, Linh M Tran, Jonathan Nakashima, Kathleen B Smith, Brenna M Tam, Yunfeng Li, Sarah M Dry, Noah Federman, William D Tap, Hong Wu, Fritz C Eilber

**Affiliations:** Department of Surgery, Division of Surgical Oncology, University of California - Los Angeles, 10833 Le Conte Ave, Room 54-140 CHS, 90095-1782 Los Angeles, California USA; Department of Molecular and Medical Pharmacology, University of California - Los Angeles, 650 Charles E Young Drive South, Room 33-131 CHS, 90095 Los Angeles, California USA; Institute for Molecular Medicine, University of California - Los Angeles, 650 Charles E Young Drive South, Room 33-257 CHS, 90095-1782 Los Angeles, California USA; Department of Pathology and Laboratory Medicine, University of California - Los Angeles, 10833 Le Conte Ave, Room 13-145D CHS, 90095 Los Angeles, California USA; Department of Pediatrics, University of California - Los Angeles, Box 951752, A2-410 MDCC, 90095 Los Angeles, California USA; Melanoma/Sarcoma Service, Division of Solid Tumors, Memorial Sloan-Kettering Cancer Center, 300 East 66th Street, 10065 New York, New York USA

**Keywords:** MPNST, Neurofibromatosis, Meta-analysis, Epidemiology

## Abstract

**Background:**

Gender-based differences in disease onset in murine models of malignant peripheral nerve sheath tumor (MPNST) and in patients with Neurofibromatosis type-1-(NF-1)-associated or spontaneous MPNST has not been well studied.

**Methods:**

Forty-three *mGFAP-Cre*+;*Pten*^*loxp*/+^;*LSL-K-ras*^*G12D*/+^ mice were observed for tumor development and evaluated for gender disparity in age of MPNST onset. Patient data from the prospectively collected UCLA sarcoma database (1974–2011, n = 113 MPNST patients) and 39 published studies on MPNST patients (n = 916) were analyzed for age of onset differences between sexes and between NF-1 and spontaneous MPNST patients.

**Results:**

Our murine model showed gender-based differences in MPNST onset, with males developing MPNST significantly earlier than females (142 vs. 162 days, p = 0.015). In the UCLA patient population, males also developed MPNST earlier than females (median age 35 vs. 39.5 years, p = 0.048). Patients with NF-1-associated MPNST had significantly earlier age of onset compared to spontaneous MPNST (median age 33 vs. 39 years, p = 0.007). However, expanded analysis of 916 published MPNST cases revealed no significant age difference in MPNST onset between males and females. Similar to the UCLA dataset, patients with NF-1 developed MPNST at a significantly younger age than spontaneous MPNST patients (p < 0.0001, median age 28 vs. 41 years) and this disparity was maintained across North American, European, and Asian populations.

**Conclusions:**

Although our preclinical model and single-institution patient cohort show gender dimorphism in MPNST onset, no significant gender disparity was detected in the larger MPNST patient meta-dataset. NF-1 patients develop MPNST 13 years earlier than patients with spontaneous MPNST, with little geographical variance.

**Electronic supplementary material:**

The online version of this article (doi:10.1186/1471-2407-14-827) contains supplementary material, which is available to authorized users.

## Background

The significance of gender as a fundamental variable to be studied in the development and progression of disease has been a long standing topic of interest [1]. Men and women differ in their genetic milieu and environmental exposures, which is reflected in overall disease susceptibilities and progression [2]. Epidemiologic studies of cancer patients reveal significant discrepancies in cancer incidence between sexes beyond the typical sex-specific malignancies [3]. For example, gastric, esophageal, brain, liver, head and neck cancers, and non-Hodgkin’s lymphoma occur more often in men than women [3–5].

Mesenchymal glioblastoma multiforme (GBM), which is thought to stem from similar biomolecular pathways as malignant peripheral nerve sheath tumors (MPNST) including loss of neurofibromin (NF1) and TP53 function [6, 7], also shows increased prevalence of disease in men [8, 9]. A murine model of GBM formation developed from NF1 deficient mice expressing a dominant-negative form of p53 (Nf1-/- DNp53) confirmed cell-intrinsic sexual dimorphism in the malignant transformation of astrocytes to GBM, regardless of hormones or tumor environment [9].

However, gender differences in the development of MPNSTs have not specifically been examined. MPNST is an aggressive soft tissue sarcoma (STS) that accounts for up to 10 percent of all STS [10–12]. It is often associated with the autosomal dominant syndrome of Neurofibromatosis type 1 (NF-1), but arise sporadically as well. MPNSTs carry a particularly poor prognosis, with a 5-year survival of 15-45% in NF-1 patients compared to 43-75% for non-NF-1 patients [10, 13–15].

Although not all gender-based disparities are felt to be based on hormonal differences, there does appear to be a notable association amongst neurofibromas (NF), benign peripheral nerve sheath tumors that are a hallmark of neurofibromatosis, and circulating hormone levels. In NF-1 patients, MPNSTs are often found in the context of a preexisting NF. Although MPNST is felt to more commonly arise from epineural and perineural NFs such as subcutaneous and plexiform NF, the subtype of dermal NF shows considerable hormone responsiveness. Dermal NFs often appear at puberty, reportedly increase in number and size during pregnancy, and may regress after delivery, implicating a possible hormonal influence on tumor growth [16–18]. It has even been suggested that steroid hormones may be involved in the malignant transformation of neurofibroma to MPNST [19]. Investigation of Schwann cell–enriched xenografted NF-1 human dermal NF, plexiform NF, and MPNST samples demonstrated that estrogen and progesterone significantly increased the growth of MPNST in 100% and 66% of samples, respectively, and also increased growth in 25% of dermal NF tested. However, estrogen and progesterone decreased growth in 25% of xenografted plexiform NF with no effect in the remaining plexiform NF samples [20].

Recently, the development of animal models that recapitulate neurofibroma and MPNST development have allowed for investigation into the mechanisms of tumor development and malignant transformation [21–23]. Interestingly, the *N*-ethyl-*N*-nitrosurea (ENU)-induced rat model and the Nf1-/+;Tp53-/+ cis (i.e. B6-NPcis) mouse model have both shown males to develop MPNSTs at an earlier age than females, even while accounting for confounding factors such as parental gender in the B6-NPcis model [21, 24, 25]. This further underscores the possibility of sex-specific differences in MPNST age of onset.

To further extend these studies, we examined our genetically engineered murine model of MPNST for gender differences. To evaluate if this model had clinically translatable findings, we conducted an analysis of MPNST patients diagnosed and treated at the University of California, Los Angeles (UCLA) and analyzed a meta-dataset of patients from 39 published clinical MPNST studies. Our study focused on age of MPNST diagnosis, as this data is consistently reported in the published MPNST literature. Evaluation of the interval between NF onset to MPNST development was not assessed, as this data is not routinely recorded. The aims of this study were to evaluate gender dimorphism in MPNST age of onset in the UCLA patient population and in a larger dataset across several geographical populations, and to evaluate differences in age of MPNST onset between NF-1 and spontaneous patients.

## Methods

### Animals

Forty-three *mGFAP-Cre*+;*Pten*^*loxp*/+^;*LSL-K-ras*^*G12D*/+^ MPNST mice were generated as previously described [21]. These mice develop multiple NFs with subsequent progression to MPNST at a reproducible rate with 100% penetrance. These mice were further purposed for MPNST chemotherapy studies (unpublished data), therefore non-invasive methods of tumor detection were used. Given the small tumor size at the time of detection, micro-PET/CT could not reliably distinguish NF from MPNST based on SUVmax (unpublished data), and was therefore not employed in this study. Physical examination was performed daily to detect tumor formation or illness. Mice were thoroughly examined and palpated for tumor formation, which consistently detected tumors as small as 4 mm diameter. We previously showed that our murine model (Gregorian et al. [19]) consistently progresses from neurofibroma to MPNST two weeks after tumor onset, regardless of gender. Therefore, initial time of palpable tumor development was used as a surrogate for MPNST development. Mice were euthanized per protocol if tumors reached a diameter of 1.5 cm or interfered with feeding, grooming or ambulation, or if the mouse lost >10% of their body weight. Survival times were not evaluated as the mice were purposed for treatment studies (unpublished). Tumors were fixed and embedded in paraffin, then sectioned and stained with hematoxylin and eosin to confirm pathologic diagnosis of MPNST. Animals were housed in a temperature-, humidity-, and light-controlled room (12-h light/dark cycle), and allowed free access to food and water. All experiments were conducted according to the research guidelines of the University of California, Los Angeles (UCLA) Chancellor’s Animal Research Committee.

### UCLA data and patient selection

Since 1974, UCLA has prospectively maintained a sarcoma database with complete clinical and pathologic patient data. A protocol detailing the study design and analysis was approved by the UCLA Institutional Review Board. For inclusion, subjects were required to have tissue diagnosis of a MPNST and undergone surgery and treatment at UCLA. 113 UCLA patients were eligible for study. Original surgical specimens were reviewed by a UCLA sarcoma pathologist (S.M.D.) to re-confirm pathologic diagnosis and grading.

### External patient database

To validate our findings within a broader dataset, we performed a PubMed search using the following algorithm “(peripheral nerve sheath tumour[Title]) OR peripheral nerve sheath tumor[Title]) OR MPNST[Title]) OR neurogenic sarcoma[Title]) OR malignant schwannoma[Title]) OR atypical neurofibromas[Title]) OR peripheral nerve sheath tumors[Title]) OR peripheral nerve sheath tumours[Title]) NOT case report[Title]” for published articles from 1960–2011. For this study, only references written in the English language were used. Studies evaluating Neurofibromatosis type 2 were excluded. 990 hits were collected, and abstracts browsed to identify relevant articles. The full manuscripts were reviewed to identify studies that listed individual patients with age of diagnosis, NF-1 status, gender, and geographic data. Ten PubMed suggested articles or citations outside of our original search were included, and met the above criteria. A total of 39 published studies with data on 916 patients with MPNST was collected and aggregated to form the meta-dataset (Additional file 1: Table S1). Data was cross-referenced to ensure duplicate patient reports were excluded. Patients were classified by geographic location, gender, and NF-1 disease status for subgroup analysis.

### Statistical analysis

Statistical significance was determined using Wilcoxon rank sum test, with a significance threshold of alpha ≤0.05. Variables assessed were age of diagnosis, gender, and NF-1 status. Analysis of the datasets was performed using STATA 12.0 (StataCorp. 2011) and R software (R-package version 2.13).

## Results

### Genetically engineered murine model of MPNST demonstrates gender dimorphism in disease onset

**Figure 1:**
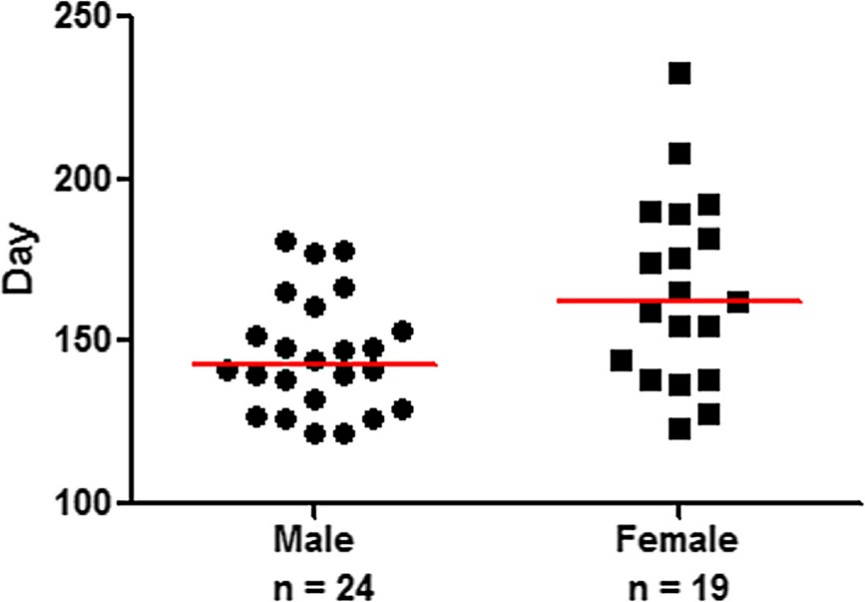
**Gender dimorphism in age of MPNST onset in genetically engineered murine model.** Female mice (black squares) developed MPNST 20 days later than male mice (black circles) (p = 0.015). Gray line symbolizes median age of diagnosis for each cohort.

In our previous study, we developed a murine neurofibroma model by conditional deletion of one allele of the *Pten* tumor suppressor gene and activation of the *K-ras*^*G12D*^ oncogene in the Schwann progenitor cells (driven by the *mGFAP-Cre* line) [21]. All neurofibroma lesions then progress to MPNST with loss of heterozygosity of the second allele of *Pten* and acquisition of high FDG-PET uptake, reminiscent of human NF-to-MPNST malignant transformation [21, 26]. To examine whether MPNST onset in this model is influenced by gender, we followed 43 *mGFAP-Cre*^+^;*Pten*^*loxp*/+^;*LSL-K-ras*^*G12D*/+^ littermates, 24 males and 19 females, for MPNST development. Mice developed MPNST at a median time of 155 days. In the context of gender-specific differences in tumor development, a significant disparity in age of MPNST onset was identified. Male mice developed MPNST at a significantly earlier age than female mice (142 versus 162 days, respectively p = 0.015, see Figure 1). In the murine ENU-induced MPNST model, genetic loci influential in female specific resistance to MPNST development were homologous to regions encoding estrogen receptors (Additional file 1: Figure S1).

### UCLA patients demonstrate gender dimorphism in age of MPNST onset

**Figure 2:**
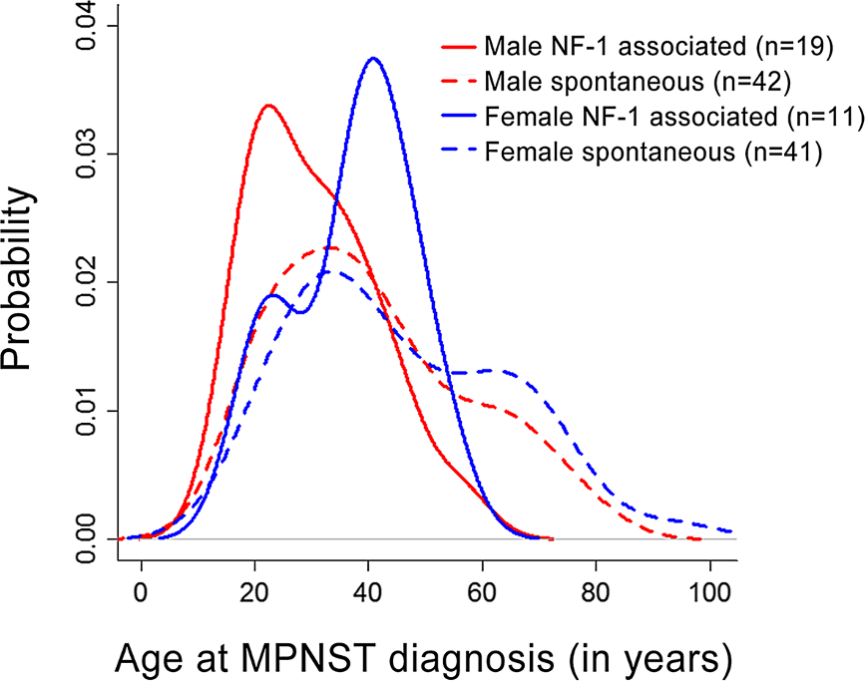
**Gender disparity in age of MPNST initial diagnosis in UCLA cohort.** Dashed lines represent spontaneous MPNST cases. Male patients are represented in red, female patients are represented in blue.

The gender dimorphism observed in the rat and mouse MPNST models prompted us to examine the age of MPNST onset in our UCLA cohort of 113 unique MPNST patients. The average age at initial MPNST diagnosis was 40 years (median 38 years, range 16–94 years). Sixty-one patients (54%) were male, and 52 patients (46%) were female. Thirty patients (26%) developed MPNST in the context of NF-1, and 83 patients (74%) developed MPNST spontaneously.Evaluation of age of MPNST diagnosis by gender revealed a trend toward males developing MPNST at an earlier age than females (Figure 2; median age 35 years versus 39.5 years, p = 0.048). Analysis of the NF-1-associated and spontaneous MPNST patient populations individually revealed no statistically significant gender differences in age of MPNST onset (p = 0.09 and p = 0.25, respectively). Interestingly, bimodal distribution was observed in each subgroup (Figure 2), and was most pronounced for female patients.

### Meta-dataset fails to show gender dimorphism in MPNST onset

**Figure 3:**
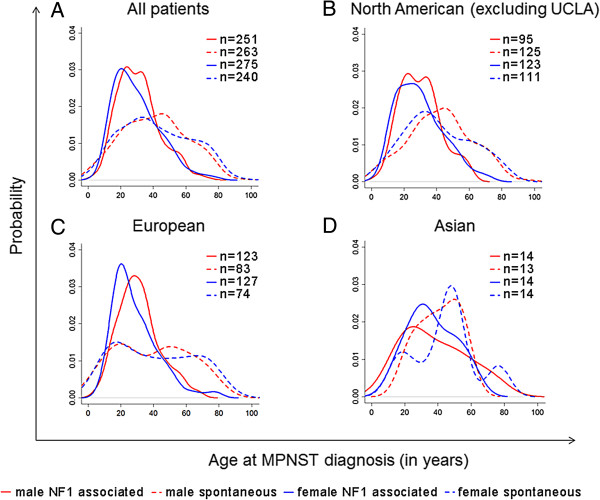
**Age of MPNST diagnosis.** Distribution of age at MPNST diagnosis in **(A)** all MPNST patients including UCLA **(B)** North American cohort **(C)** European cohort **(D)** Asian cohort. Spontaneous MPNST patients are depicted with a dashed line, NF-1 associated MPNST patients are depicted with a solid line. Males are represented in red, females are represented in blue.

To test whether the gender dimorphism observed in a small cohort of UCLA patients could be confirmed in a larger MPNST population, we conducted an analysis based on publically available datasets. The meta-dataset of patients with MPNST included North American, European, and Asian populations; 916 total unique patients were identified. The overall median age at MPNST diagnosis for men was 34 years (n = 451), compared to the median age of 31 years in females (n = 465) however this discrepancy was not significant (p = 0.26; See Table 1, Figure 3A). Subgroup analysis between males and females in the NF-1 population and in the spontaneous population showed no significant difference in age of diagnosis (Figure 3A; p = 0.31 and p = 0.99, respectively).

**Table 1:** **UCLA and meta-dataset analysis shows significantly earlier age of MPNST onset in NF-1 patients compared to spontaneous MPNST patients**

Dataset	Disease	Male median age (n)	Female median age (n)	Male vs. female Wilcox p value	Overall median age (n)	NF-1 vs. spont. Wilcox p value
UCLA	NF-1	31 yrs (19)	39 yrs (11)	0.09	33 yrs (30)	0.007
	Spont.	37 yrs (42)	40 yrs (41)	0.25	39 yrs (83)	
Others	NF-1	29 yrs (230)	27 yrs (266)	0.11	28 yrs (496)	<0.0001
	Spont.	43 yrs (221)	39 yrs (199)	0.88	41 yrs (420)

### NF-1 associated MPNST patients develop MPNST at a significantly earlier age than spontaneous MPNST patients

Further analysis of the UCLA dataset revealed that patients with NF-1-associated MPNST had a significantly earlier age of onset compared to patients with spontaneous MPNST (Figure 2; median age 33 years vs. 39 years, p = 0.007). In the UCLA dataset, patients with NF-1 developed MPNST an average of 10.3 years earlier than patients without NF-1.Similar to the UCLA dataset, the meta-dataset indicated that patients with NF-1 developed MPNST at a significantly younger age than patients without NF-1 (median 28 vs. 41 years old, p < 0.0001). Evaluation of the overall distribution of male and female age of onset (Figure 3A) revealed distinct peaks for NF-1 associated MPNST onset, as compared to the more broad distribution seen in spontaneous MPNST diagnosis.The data was then analyzed by geographical regions of North America, Europe, and Asia. In the North American population, the median age of NF-1 patient MPNST diagnosis was 28 (n = 218) compared to the age of 40 in spontaneous MPNST patients (n = 236) (Figure 3B). In the European population, the median age of diagnosis in NF-1 patients was 27.9 (n = 250) compared to the spontaneous MPNST patient age of 41 (n = 157) (Figure 3C). Asian populations showed a larger disparity in age of diagnosis; however the size of this cohort was considerably smaller than the others. In Asian populations, the median age of diagnosis in NF-1 patients was 33.5 years (n = 28), compared to a median age of diagnosis of 45 years in the spontaneous MPNST population (p = 0.23) (Figure 3D).

## Discussion

Our genetically engineered murine model of MPNST showed significant gender dimorphism in age of MPNST onset. To see if this was clinically translatable, we investigated gender differences in age of MPNST onset first in our UCLA patient cohort and second in a larger meta-analysis of 916 published MPNST patients. The distribution of MPNST age of onset in our UCLA patient cohort mirrored the gender dimorphism found in our pre-clinical model, with men diagnosed with MPNST at an earlier age, however this finding was not reflected in the larger meta-analysis. The interrogation of gender in our murine model (castration, hormonal manipulation, etc.) was not pursued further as it was not replicated in the larger patient meta-dataset. The gender disparity in the UCLA patient population is likely due to the relative rarity of MPNST, as our MPNST patient population was relatively small (n = 113). The limitation of this initial result underscores the need for collaborative effort in the study of STS subtypes. In rare cancers, such as sarcoma, collective pursuits of clinical research should be encouraged to increase the power and effect-size of the analysis. When our initial result was tested in a larger “meta-dataset” cohort, we believe a clearer picture of MPNST onset was revealed and gender-based differences were not observed.

Additionally, recent literature supports multidisciplinary management of patients with STS at a nationally recognized sarcoma center. Age of diagnosis is likely influenced by non-clinical factors such as adherence to guidelines, referral pathways, and time to referral to a sarcoma center, which were not analyzed in this study. Earlier age of MPNST diagnosis is likely favored by prompt referral to a sarcoma center, and should therefore be encouraged in patients with suspicious tumors.

Analysis of the meta-dataset from 39 published studies on MPNST revealed a 13 year difference in median age of diagnosis between NF-1 and spontaneous MPNST patients. Using a larger multinational meta-dataset of 916 patients, we were able to confirm the findings of earlier reports on smaller cohorts [10, 27]. Given the rarity of MPNST, it is important to study clinicopathologic disease characteristics in larger, adequately powered cohorts when possible. The earlier reports of age of onset differences were based on geographically localized, single-institutional, small populations whose results had the potential to be skewed by institutional differences in monitoring and diagnosis protocols [10, 27]. The disparity in age of onset in our meta-dataset was maintained in North American, European, and Asian populations. In the combined meta-dataset, the age of onset for the NF-1 associated MPNST patients was centered around a distinct peak, which differed significantly from the broad distribution of age at diagnosis for spontaneous cases that spans decades. This wide variance in spontaneous cases suggests that the underlying genetic cause of spontaneous MPNST formation may be the accumulation of a wide assortment of genetic aberrations over time, in contrast to NF-1 MPNST formation which may be due to a few specific genetic mutations.

This disparity between NF-1 and spontaneous patients may also reflect the earlier and increased frequency of routine physical exams and surveillance for MPNSTs in NF-1 patients. Clinical manifestations of malignant transformation classically rely on reported symptoms of new neurological deficits, rapid increase in size, change in palpated density from soft to hard, and unremitting pain. Patients with NF-1 and clinicians who treat them are often aware of the 10% lifetime risk of MPNST, and might be more apt to notice these changes. The malignant transformation event, whereby a NF degenerates into a MPNST, is distinct from the time at which a patient is formally diagnosed with MPNST. The interval between MPNST development and diagnosis can be shortened with the use of FDG-PET, as it has emerged as an accurate method to distinguish NF from MPNST based on the glycolytic phenotype [26]. Using quantitative SUV_max_ measurements, the gap in time between actual MPNST development and formal diagnosis can be truncated.

Limitations of this study are ones inherent in studying a rare disease. First, patient diagnosis from both our UCLA and meta-datasets spans decades, covering a time period in which the diagnostic tools for MPNST detection were greatly improved. Improved detection through computerized tomography and magnetic resonance imaging has likely led to earlier detection of MPNST. However, for the same time period, this would affect patients of either sex equally, and affect both NF-associated and spontaneous MPNST patients equally. Second, there may be reporting bias, as most datasets were from surgically-resected patients only, and exclude patients with biopsy-proved but unresectable MPNST.

This study focused on MPNST age of onset only, and did not include analysis of clinical factors such as age of NF-1 diagnosis or disease stage at MPNST diagnosis, due to lack of data reporting in the majority of the studies used in the meta-analysis. Patient gender may influence the development and progression of MPNST, however reports are conflicting. A recent analysis by Kolberg et al. [10], found no survival advantage between males and females (in 117 spontaneous patients and 62 NF-1 patients) [14]. However, a separate study by Ingham et al. [21] found a significant difference in survival between male and female MPNST patients, with men having worse survival (n = 52, p = 0.05) [28]. Given these contradictory findings, any gender-specific survival advantage remains to be determined, and should be investigated within a larger cohort.

## Conclusion

In conclusion, the difference in age of onset between MPNST formation in NF-1 associated cases and spontaneous cases is reflected in both the actual age of onset and in the population age distribution. This suggests that the two branches of MPNST development are likely rooted in distinct and separate origins, influenced by different genetic and environmental factors.

## Electronic supplementary material

Additional file 1: **Supplemental Materials. Figure S1.** Genetic homology to rat Mss4 locus on chromosome 6q24, including human homologs ERB2 and ERBB, which encode for estrogen receptors. **Table S1.** External datasets used in this study [27, 29–66]. (DOC 247 KB)

## References

[1] *Exploring the Biological Contributions to Human Health: Does Sex Matter?*. Washington, DC: The National Academies Press; 2001. 25057540

[2] Benigni R (2007). Social sexual inequality and sex difference in cancer incidence. Environ Res.

[3] Cook MB, Dawsey SM, Freedman ND, Inskip PD, Wichner SM, Quraishi SM, Devesa SS, McGlynn KA (2009). Sex disparities in cancer incidence by period and age. Cancer Epidemiol Biomarkers Prev.

[4] McCann J (2000). Gender differences in cancer that don't make sense–or do they?. J Natl Cancer Inst.

[5] Sun T, Warrington NM, Rubin JB (2012). Why does Jack, and not Jill, break his crown? Sex disparity in brain tumors. Biol Sex Differ.

[6] Verhaak RG, Hoadley KA, Purdom E, Wang V, Qi Y, Wilkerson MD, Miller CR, Ding L, Golub T, Mesirov JP, Alexe G, Lawrence M, O'Kelly M, Tamayo P, Weir BA, Gabriel S, Winckler W, Gupta S, Jakkula L, Feiler HS, Hodgson JG, James CD, Sarkaria JN, Brennan C, Kahn A, Spellman PT, Wilson RK, Speed TP, Gray JW, Meyerson M (2010). Integrated genomic analysis identifies clinically relevant subtypes of glioblastoma characterized by abnormalities in PDGFRA, IDH1, EGFR, and NF1. Cancer Cell.

[7] Yang J, Du X (2013). Genomic and molecular aberrations in malignant peripheral nerve sheath tumor and their roles in personalized target therapy. Surg Oncol.

[8] Dubrow R, Darefsky AS (2011). Demographic variation in incidence of adult glioma by subtype, United States, 1992-2007. BMC Cancer.

[9] Sun T, Warrington NM, Luo J, Brooks MD, Dahiya S, Snyder SC, Sengupta R, Rubin JB (2014). Sexually dimorphic RB inactivation underlies mesenchymal glioblastoma prevalence in males. J Clin Invest.

[10] Ducatman BS, Scheithauer BW, Piepgras DG, Reiman HM, Ilstrup DM (1986). Malignant peripheral nerve sheath tumors. A clinicopathologic study of 120 cases. Cancer.

[11] McGaughran JM, Harris DI, Donnai D, Teare D, MacLeod R, Westerbeek R, Kingston H, Super M, Harris R, Evans DG (1999). A clinical study of type 1 neurofibromatosis in north west England. J Med Genet.

[12] Vauthey JN, Woodruff JM, Brennan MF (1995). Extremity malignant peripheral nerve sheath tumors (neurogenic sarcomas): a 10-year experience. Ann Surg Oncol.

[13] Ghosh BC, Ghosh L, Huvos AG, Fortner JG (1973). Malignant schwannoma. A Clinicopathologic Study Cancer.

[14] Kolberg M, Holand M, Agesen TH, Brekke HR, Liestol K, Hall KS, Mertens F, Picci P, Smeland S, Lothe RA (2013). Survival meta-analyses for >1800 malignant peripheral nerve sheath tumor patients with and without neurofibromatosis type 1. Neuro-Oncology.

[15] Guccion JG, Enzinger FM (1979). Malignant Schwannoma associated with von Recklinghausen's neurofibromatosis. Virchows Archiv A, Pathol Anatomy Histol.

[16] Swapp GH, Main RA (1973). Neurofibromatosis in pregnancy. Brit J Dermatol.

[17] Sharpe JCaY RH (1937). Recklinghausen's neurofibromatosis: clinical manifestations in thirty-one cases. Arch Intern Med.

[18] Roth TM, Petty EM, Barald KF (2008). The role of steroid hormones in the NF1 phenotype: focus on pregnancy. Am J Med Genet A.

[19] Fishbein L, Zhang X, Fisher LB, Li H, Campbell-Thompson M, Yachnis A, Rubenstein A, Muir D, Wallace MR (2007). In vitro studies of steroid hormones in neurofibromatosis 1 tumors and Schwann cells. Mol Carcinog.

[20] Li H, Zhang X, Fishbein L, Kweh F, Campbell-Thompson M, Perrin GQ, Muir D, Wallace M (2010). Analysis of steroid hormone effects on xenografted human NF1 tumor schwann cells. Cancer Biol Ther.

[21] Gregorian C, Nakashima J, Dry SM, Nghiemphu PL, Smith KB, Ao Y, Dang J, Lawson G, Mellinghoff IK, Mischel PS, Phelps M, Parada LF, Liu X, Sofroniew MV, Eilber FC, Wu H (2009). PTEN dosage is essential for neurofibroma development and malignant transformation. Proc Natl Acad Sci U S A.

[22] Winzen B, Koelsch B, Fischer C, Kindler-Rohrborn A (2009). Genetic basis of sex-specific resistance to neuro-oncogenesis in (BDIX x BDIV) F(2) rats. Mamm Genome.

[23] Koelsch BU, Fischer C, Neibecker M, Schmitt N, Schmidt O, Rajewsky MF, Kindler-Rohrborn A (2006). Gender-specific polygenic control of ethylnitrosourea-induced oncogenesis in the rat peripheral nervous system. Int J Cancer J Int Du Cancer.

[24] Koelsch B, Winzen-Reichert B, Fischer C, Kutritz A, van den Berg L, Kindler-Rohrborn A (2011). Sex-biased suppression of chemically induced neural carcinogenesis in congenic BDIX.BDIV-Mss4a rats. Physiol Genomics.

[25] Walrath JC, Fox K, Truffer E, Gregory Alvord W, Quinones OA, Reilly KM (2009). Chr 19(A/J) modifies tumor resistance in a sex- and parent-of-origin-specific manner. Mamm Genome.

[26] Benz MR, Czernin J, Dry SM, Tap WD, Allen-Auerbach MS, Elashoff D, Phelps ME, Weber WA, Eilber FC (2010). Quantitative F18-fluorodeoxyglucose positron emission tomography accurately characterizes peripheral nerve sheath tumors as malignant or benign. Cancer.

[27] Hruban RH, Shiu MH, Senie RT, Woodruff JM (1990). Malignant peripheral nerve sheath tumors of the buttock and lower extremity. A study of 43 cases. Cancer.

[28] Ingham SHS, Moran A, Wylie J, Leahy M, Evans DG (2011). Malignant peripheral nerve sheath tumours in NF1: improved survival in women and in recent years. Eur J Cancer.

[29] D'Agostino AN, Soule EH, Miller RH (1963). Primary malignant neoplasms of nerves (malignant neurilemomas) in patients without manifestations of multiple neurofibromatosis (Von Recklinghausen's disease). Cancer.

[30] D'Agostino AN, Soule EH, Miller RH (1963). Sarcomas of the peripheral nerves and somatic soft tissue associated with multiple neurofibromatosis (Von Recklinghausen's disease). Cancer.

[31] White HR (1971). Survival in malignant schwannoma. An 18-year study. Cancer.

[32] Trojanowski JQ, Kleinman GM, Proppe KH (1980). Malignant tumors of nerve sheath origin. Cancer.

[33] Arpornchayanon O, Hirota T, Itabashi M, Nakajima T, Fukuma H, Beppu Y, Nishikawa K (1984). Malignant peripheral nerve tumors: a clinicopathological and electron microscopic study. Jpn J Clin Oncol.

[34] Bojsen-Moller M, Myhre-Jensen O (1984). A consecutive series of 30 malignant schwannomas. Survival in relation to clinico-pathological parameters and treatment. Acta Pathol Microbiol Immunol Scand A.

[35] Ducatman BS, Scheithauer BW (1984). Malignant peripheral nerve sheath tumors with divergent differentiation. Cancer.

[36] Daimaru Y, Hashimoto H, Enjoji M (1985). Malignant peripheral nerve-sheath tumors (malignant schwannomas). An immunohistochemical study of 29 cases. Am J Surg Pathol.

[37] Bailet JW, Abemayor E, Andrews JC, Rowland JP, Fu YS, Dawson DE (1991). Malignant nerve sheath tumors of the head and neck: a combined experience from two university hospitals. Laryngoscope.

[38] de Cou JM, Rao BN, Parham DM, Lobe TE, Bowman L, Pappo AS, Fontanesi J (1995). Malignant peripheral nerve sheath tumors: the St. Jude Children's Research Hospital experience. Ann Surg Oncol.

[39] Kunisada T, Kawai A, Ozaki T, Sugihara S, Taguchi K, Inoue H (1997). A clinical analysis of malignant schwannoma. Acta Med Okayama.

[40] Angelov L, Davis A, O'Sullivan B, Bell R, Guha A (1998). Neurogenic sarcomas: experience at the University of Toronto. Neurosurgery.

[41] Kourea HP, Bilsky MH, Leung DH, Lewis JJ, Woodruff JM (1998). Subdiaphragmatic and intrathoracic paraspinal malignant peripheral nerve sheath tumors: a clinicopathologic study of 25 patients and 26 tumors. Cancer.

[42] Casanova M, Ferrari A, Spreafico F, Luksch R, Terenziani M, Cefalo G, Massimino M, Gandola L, Lombardi F, Fossati-Bellani F (1999). Malignant peripheral nerve sheath tumors in children: a single-institution twenty-year experience. J Pediatr Hematol Oncol.

[43] Liapis H, Marley EF, Lin Y, Dehner LP (1999). p53 and Ki-67 proliferating cell nuclear antigen in benign and malignant peripheral nerve sheath tumors in children. Pediatr Dev Pathol.

[44] Schmidt H, Taubert H, Meye A, Wurl P, Bache M, Bartel F, Holzhausen HJ, Hinze R (2000). Gains in chromosomes 7, 8q, 15q and 17q are characteristic changes in malignant but not in benign peripheral nerve sheath tumors from patients with Recklinghausen's disease. Cancer Lett.

[45] Schmidt H, Wurl P, Taubert H, Meye A, Bache M, Holzhausen HJ, Hinze R (1999). Genomic imbalances of 7p and 17q in malignant peripheral nerve sheath tumors are clinically relevant. Genes Chromosomes Cancer.

[46] Ferner RE, Lucas JD, O'Doherty MJ, Hughes RA, Smith MA, Cronin BF, Bingham J (2000). Evaluation of (18)fluorodeoxyglucose positron emission tomography ((18)FDG PET) in the detection of malignant peripheral nerve sheath tumours arising from within plexiform neurofibromas in neurofibromatosis 1. J Neurol Neurosurg Psychiatry.

[47] Mertens F, Dal Cin P, De Wever I, Fletcher CD, Mandahl N, Mitelman F, Rosai J, Rydholm A, Sciot R, Tallini G, van Den Berghe H, Vanni R, Willén H (2000). Cytogenetic characterization of peripheral nerve sheath tumours: a report of the CHAMP study group. J Pathol.

[48] Leroy K, Dumas V, Martin-Garcia N, Falzone MC, Voisin MC, Wechsler J, Revuz J, Creange A, Levy E, Lantieri L, Zeller J, Wolkenstein P (2001). Malignant peripheral nerve sheath tumors associated with neurofibromatosis type 1: a clinicopathologic and molecular study of 17 patients. Arch Dermatol.

[49] Evans DG, Baser ME, McGaughran J, Sharif S, Howard E, Moran A (2002). Malignant peripheral nerve sheath tumours in neurofibromatosis 1. J Med Genet.

[50] Zhou H, Coffin CM, Perkins SL, Tripp SR, Liew M, Viskochil DH (2003). Malignant peripheral nerve sheath tumor: a comparison of grade, immunophenotype, and cell cycle/growth activation marker expression in sporadic and neurofibromatosis 1-related lesions. Am J Surg Pathol.

[51] Watson MA, Perry A, Tihan T, Prayson RA, Guha A, Bridge J, Ferner R, Gutmann DH (2004). Gene expression profiling reveals unique molecular subtypes of Neurofibromatosis Type I-associated and sporadic malignant peripheral nerve sheath tumors. Brain Pathol.

[52] Tucker T, Wolkenstein P, Revuz J, Zeller J, Friedman JM (2005). Association between benign and malignant peripheral nerve sheath tumors in NF1. Neurology.

[53] Brenner W, Friedrich RE, Gawad KA, Hagel C, von Deimling A, de Wit M, Buchert R, Clausen M, Mautner VF (2006). Prognostic relevance of FDG PET in patients with neurofibromatosis type-1 and malignant peripheral nerve sheath tumours. Eur J Nucl Med Mol Imaging.

[54] Upadhyaya M, Spurlock G, Majounie E, Griffiths S, Forrester N, Baser M, Huson SM, Gareth Evans D, Ferner R (2006). The heterogeneous nature of germline mutations in NF1 patients with malignant peripheral serve sheath tumours (MPNSTs). Hum Mutat.

[55] Holtkamp N, Atallah I, Okuducu AF, Mucha J, Hartmann C, Mautner VF, Friedrich RE, Mawrin C, von Deimling A (2007). MMP-13 and p53 in the progression of malignant peripheral nerve sheath tumors. Neoplasia.

[56] Minovi A, Basten O, Hunter B, Draf W, Bockmuhl U (2007). Malignant peripheral nerve sheath tumors of the head and neck: management of 10 cases and literature review. Head Neck.

[57] Tabone-Eglinger S, Bahleda R, Cote JF, Terrier P, Vidaud D, Cayre A, Beauchet A, Theou-Anton N, Terrier-Lacombe MJ, Lemoine A, Penault-Llorca F, Le Cesne A, Emile JF (2008). Frequent EGFR Positivity and Overexpression in High-Grade Areas of Human MPNSTs. Sarcoma.

[58] Upadhyaya M, Kluwe L, Spurlock G, Monem B, Majounie E, Mantripragada K, Ruggieri M, Chuzhanova N, Evans DG, Ferner R, Thomas N, Guha A, Mautner V (2008). Germline and somatic NF1 gene mutation spectrum in NF1-associated malignant peripheral nerve sheath tumors (MPNSTs). Hum Mutat.

[59] Brekke HR, Kolberg M, Skotheim RI, Hall KS, Bjerkehagen B, Risberg B, Domanski HA, Mandahl N, Liestol K, Smeland S, Danielsen HE, Mertens F, Lothe RA (2009). Identification of p53 as a strong predictor of survival for patients with malignant peripheral nerve sheath tumors. Neuro-Oncology.

[60] Brekke HR, Ribeiro FR, Kolberg M, Agesen TH, Lind GE, Eknaes M, Hall KS, Bjerkehagen B, van den Berg E, Teixeira MR, Mandahl N, Smeland S, Mertens F, Skotheim RI, Lothe RA (2010). Genomic changes in chromosomes 10, 16, and X in malignant peripheral nerve sheath tumors identify a high-risk patient group. J Clin Oncol.

[61] Rekhi B, Ingle A, Kumar R, DeSouza MA, Dikshit R, Jambhekar NA (2010). Malignant peripheral nerve sheath tumors: clinicopathological profile of 63 cases diagnosed at a tertiary cancer referral center in Mumbai. India Indian J Pathol Microbiol.

[62] Subramanian S, Thayanithy V, West RB, Lee CH, Beck AH, Zhu S, Downs-Kelly E, Montgomery K, Goldblum JR, Hogendoorn PC, Corless CL, Oliveira AM, Dry SM, Nielsen TO, Rubin BP, Fletcher JA, Fletcher CD, van de Rijn M (2010). Genome-wide transcriptome analyses reveal p53 inactivation mediated loss of miR-34a expression in malignant peripheral nerve sheath tumours. J Pathol.

[63] Beert E, Brems H, Daniels B, De Wever I, Van Calenbergh F, Schoenaers J, Debiec-Rychter M, Gevaert O, De Raedt T, Van Den Bruel A, de Ravel T, Cichowski K, Kluwe L, Mautner V, Sciot R, Legius E (2011). Atypical neurofibromas in neurofibromatosis type 1 are premalignant tumors. Genes Chromosomes Cancer.

[64] Pryor JG, Brown-Kipphut BA, Iqbal A, Scott GA (2011). Microarray comparative genomic hybridization detection of copy number changes in desmoplastic melanoma and malignant peripheral nerve sheath tumor. Am J Dermatopathol.

[65] Yang J, Ylipaa A, Sun Y, Zheng H, Chen K, Nykter M, Trent J, Ratner N, Lev DC, Zhang W (2011). Genomic and molecular characterization of malignant peripheral nerve sheath tumor identifies the IGF1R pathway as a primary target for treatment. Clin Cancer Res.

[66] Yu J, Deshmukh H, Payton JE, Dunham C, Scheithauer BW, Tihan T, Prayson RA, Guha A, Bridge JA, Ferner RE, Lindberg GM, Gutmann RJ, Emnett RJ, Salavaggione L, Gutmann DH, Nagarajan R, Watson MA, Perry A (2011). Array-based comparative genomic hybridization identifies CDK4 and FOXM1 alterations as independent predictors of survival in malignant peripheral nerve sheath tumor. Clin Cancer Res.

[CR67] The pre-publication history for this paper can be accessed here:http://www.biomedcentral.com/1471-2407/14/827/prepub

