# Supplementary material for: Gender dimorphism and age of onset in malignant peripheral nerve sheath tumor preclinical models and human patients
Source: BMC Cancer. 2014 Nov 15;14:827. doi: 10.1186/1471-2407-14-827 (PMC4237782; doi:10.1186/1471-2407-14-827)
Supplement: Supplementary file 1 — Additional file 1: Supplemental Materials. Figure S1. Genetic homology to rat Mss4 locus on chromosome 6q24, including human homologs ERB2 and ERBB, which encode for estrogen receptors. Table S1. External datasets used in this study [27, 29–66]. (DOC 247 KB) [file 12885_2013_5006_MOESM1_ESM.doc]

Supplemental Materials:

*Evaluation of genetic homologs possibly influential in gender dimorphism.*

Kindler-Rohrborn and colleagues[^17^](#_ENREF_17) identified homozygous BDIV alleles at two loci Mss4 (chromosome 6) and Mss7 (chromosome 10) that increase resistance of N-ethyl-N-nitrosourea (ENU) induced MPNST development in female rats. In this analysis we used the Rat Genome Browser (http://rgd.mcw.edu/wg/tool-menu) and the UCSB genome browser with BLAST E-values<1x10^-15^ to search for homologs on murine and human chromosomes that correspond to these gender associated loci.

The rat Mss4 locus on 6q24 is homologous to mouse 12qC2-qD3. However, in the human genome, this locus is mapped to two distinct regions on chromosome 14: 14q21.3-q22.1 and 14q23.1-24.3 (Supplemental Figure 1). Interestingly, this region contains genes encoding estrogen receptors. The genes ESR2 (estrogen receptor beta) and ESRRB (estrogen-related receptor beta) were among the homologous genes identified in our genetic study.


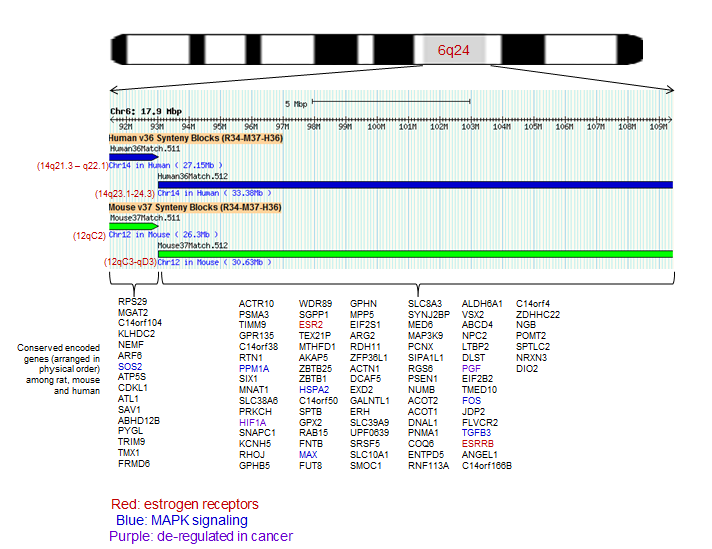
Supplemental Figure 1. Genetic homology to rat Mss4 locus on chromosome 6q24, including human homologs ERB2 and ERBB, which encode for estrogen receptors.

Supplemental Table S1. External datasets used in this study.

| Dataset | First author, publication year | State, Country | Number of Patients | |
| --- | --- | --- | --- | --- |
|  |  |  | NF1 | spontaneous |
| 1 | D’Agostino, 1963 | MN, USA | 0 | 24 |
| 2 | D’Agostino, 1963 | MN, USA | 21 | 0 |
| 3 | White, 1971 | VA, USA | 10 | 5 |
| 4 | Trojanowski, 1980 | MA, USA | 2 | 16 |
| 5 | Arpornchayanon, 1984 | Tokyo, Japan | 2 | 14 |
| 6 | Bojsen-Møller, 1984 | Aarhus, Denmark | 8 | 22 |
| 7 | Ducatman, 1984 | MN, USA | 9 | 5 |
| 8 | Daimaru, 1985 | Fukuoka, Japan | 11 | 7 |
| 9 | Hruban, 1990 | NY, USA | 23 | 20 |
| 10 | Bailet, 1991 | CA, USA | 0 | 16 |
| 11 | deCou, 1995 | TN, USA | 11 | 17 |
| 12 | Kunisada, 1997 | Okayama, Japan | 5 | 6 |
| 13 | Angelov, 1998 | Ontario, Canada | 7 | 11 |
| 14 | Kourea, 1998 | NY, USA | 15 | 10 |
| 15 | Casanova, 1999 | Milano, Italy | 7 | 17 |
| 16 | Liapis, 1999 | MO, USA | 6 | 4 |
| 17 | Schmidt, 1999 and 2000 | Hale, Germany | 6 | 20 |
| 18 | Ferner, 2000 | London, UK | 3 | 0 |
| 19 | Mertens, 2000 | Belgium | 4 | 8 |
| 20 | Leroy, 2001 | France | 17 | 0 |
| 21 | Evans, 2002 | Manchester, UK | 32 | 0 |
| 22 | Zhou, 2003 | UT, USA | 13 | 7 |
| 23 | Watson, 2004 | Multiple centers in UK and USA | 25 | 17 |
| 24 | Tucker, 2005 | France | 25 | 0 |
| 25 | Brenner, 2006 | Hamburg, Germany | 16 | 0 |
| 26 | Upadhyaya, 2006 | UK | 30 | 0 |
| 27 | Holtkamp, 2007 | Germany | 22 | 14 |
| 28 | Minovi, 2007 | Fulda, Germany | 1 | 6 |
| 29 | Tabone-Eglinger, 2008 | France | 26 | 26 |
| 30 | Upadhyaya, 2008 | UK | 15 | 0 |
| 31 | Brekke, 2009 and 2010 | Multiple centers in Europe | 38 | 44 |
| 32 | Rekhi, 2010 | India | 10 | 0 |
| 33 | Subramanian, 2010 | Multiple centers in USA | 11 | 3 |
| 34 | Beert, 2011 | Multiple centers in Europe and USA | 8 | 15 |
| 35 | Pryor, 2011 | NY, USA | 3 | 2 |
| 36 | Yang, 2011 | TX, USA | 13 | 10 |
| 37 | Yu, 2011 | MO, USA | 19 | 13 |
